# The Brain of the Negro Compared with That of the European and the Orang-Outang

**Published:** 1839-10

**Authors:** 


### Art. IV.

*Das Hirn des Negers, mit dem des Europäers und Orang-Outangs verglichen.* Von Dr. F. Tiedemann. *Mit sechs Tafeln.—Heidelberg,* 1837. 4to, pp. 84.

*The Brain of the Negro compared with that of the European and the Orang-Outang.* By Dr. F. Tiedemann. *With six plates.—Heidelberg,* 1837.

The relative capabilities of the Negro and the European races of mankind for intellectual and moral improvement—a question in itself of the highest interest, from its intrinsic value, as elucidating the natural history of the human species—has of late years acquired additional importance, from the fact that it is now upon the eve of being withdrawn from the regions of mere speculative enquiry, and subjected to the severely-searching test of actual experience. That opinions upon this point should yet remain so much divided, notwithstanding the numerous and varied investigations which it has undergone, is a sufficient proof of the great practical difficulties in which the subject is naturally involved. At the same time it must be confessed that these difficulties have been materially increased by the prejudiced views so industriously spread abroad by those whose interest it is or seemed to be to degrade the unfortunate negro as much as possible. For although, in the present state of general information, any attempt to represent the negro as merely a connecting link between the human species and the ape, as maintained by Monboddo, Rousseau, and White, would scarcely be received with any other feeling than that of ridicule, yet it cannot be denied that the exaggerated statements of his intellectual and moral inferiority, his unconquerable indolence, and the inherent treachery of his disposition, which have been thus widely circulated, have excited in the minds of many considerable doubts as to the wisdom of the recent measures of emancipation, and no slight degree of fear for the safety of the white settlers. It is therefore with great satisfaction that we hail any attempts to place the question in a clearer light, and more especially when, as in the work before us, the principles of philosophical induction are applied to its elucidation.

The monograph whose title stands at the head of these remarks is an extension and amplification of a paper by the same learned author, which was read before the Royal Society of London, June 9th, 1836. Of this paper we formerly gave a very brief notice, (*Brit. and For. Med. Rev.*, vol. IV. p. 529;) and in our number for April, 1838, we inserted a few observations by Dr. Combe, extracted from the Phrenological Journal, and calculated to throw strong doubts upon the accuracy of the inferences drawn by the author. But the great interest and importance of the several topics embraced in this enquiry have induced us to believe that a more extended investigation into its merits would not be without value; and we propose, therefore, in the following pages, to enter upon a detailed examination of the anatomical facts brought forward, and the inferences which have been deduced from them, making, as we proceed, such remarks as may appear to us necessary, either to confirm or refute the statements in the text.

Our author endeavours to determine the much litigated point of the Negro's intellectual and moral capacity, by following out a hitherto untried method, viz. by a comparison of the relative size and weight of the brain in the European, the Negro, and the orang-outang; and, by enquiring into the existence or non-existence of differences (if any) in their external appearance and internal structure, he attempts in this way to answer the two following questions: 1st. Is the brain of the Negro in any material respect different from that of the European? 2d. Does the brain of the Negro bear more resemblance to that of the orang-outang than the European?

Abundant materials for this enquiry were afforded him by numerous observations already carried on during a series of years upon the brains and skulls of individuals of all races of mankind, and by frequent admeasurement of the same parts in the orang; the most celebrated anatomical museums both on the continent and in Great Britain having been consulted for this purpose.

I. *Weight of the Brain of Europeans.* But little attention has, until lately, been paid to the determination of the size and weight of the human brain, the older anatomists almost uniformly contenting themselves with coinciding in the dogma laid down by the Greek physicians. that the brain of man is larger than that of any other animal. Piccoluomini appears to have been the first who attempted to compute its weight, which he states as varying from four to five pounds. Succeeding observers have given very different estimates; but they are all of little value, because the scale of measurement is not specified in most instances (comparison being thus rendered impossible), and because no distinction has been made regarding the age, sex, stature, and condition of each person examined. The observations of Sir W. Hamilton and Dr. Sims were conducted with greater care, and are more worthy of confidence, but they too differ considerably in their results; and it may be remarked of all these estimates, and of those also which were obtained by Tiedemann, that one source of fallacy has been uniformly overlooked, viz. the influence which various diseases exert over the weight of the encephalon, as has been most clearly shown by the very valuable results of Dr. Clendinning's experiments, recorded in his Croonian Lectures for 1838 (vide *Lond. Med. Gaz.*) "An increase of weight in the encephalon," he remarks, "is an usual effect or concomitant of morbus cordis;" and when we consider the frequency of affections of this nature, either idiopathic or as complications of other diseases, we shall see at once that any conclusions drawn from cases in which these distinctions are not noticed, cannot be entirely relied upon, however accurate they may be in other respects. Professor Tiedemann himself, in a subsequent part of his essay, acknowledges that the weight of the brain is altered by diseases; and yet in his tables he has altogether neglected this important fact. We proceed, however, with our author's investigations.

His object being to ascertain both the absolute weight of the brain and its relation to that of the whole body, numerous enquiries were instituted upon persons of both sexes, from birth up to the 82d year, great attention being in most instances paid to the stature, weight, and state of nutrition of the individual. The brain was always separated from the spinal cord, at the part where the pyramidal bodies, after their decussation, terminate in the medulla oblongata. The nerves were divided at their entrance into the foramina at the base of the cranium, and the arachnoid and pia mater were then carefully removed.

The first two tables exhibit the results obtained from the examination of the brains of 47 males and 18 females, from which the following inferences are deduced : Of 39 males, between the ages of twenty-two and eighty-two, the weight of the brain varied from 3lb. 2oz. 20gr. to 4lb. 11oz. 4dr. Among these 39, in 14 the weight of the brain exceeded 4lbs. These estimates, it may be observed, are considerably above those of Dr. Sims, who proved that in 70 cases the majority varied between 3lb. 8oz. 1dr. 20gr., and 3lb. 11oz. 6dr.; and that 13 only had a brain weighing more than 4lbs. Of 11 females, from twenty to eighty years of age, the weight varied between 2lb. 8oz. 5dr. 50gr. and 3lb. 10oz. 2dr., thus showing that the female brain has in general a much less weight than the male, a fact which is in accordance with the observations of Sir W. Hamilton, who remarks that in 100 cases scarcely one will be found weighing 4lb. troy. This difference exists even at the time of birth, the brain of new-born males weighing on an average from 13 to 14oz., that of new-born females from 9 to 12oz.

Various opinions have been adopted by anatomists regarding the period at which the brain attains its greatest size. Sœmmerring placed it in the third year ; Gall and Spurzheim about the fortieth ; while Sims believed that he had ascertained what, if true, would be a most singular fact—that its growth continued until the twentieth year ; that from this to the thirtieth the brain diminished in bulk, then increased again until it attained its maximum size, between the fortieth and fiftieth year, after which period it once more gradually declined. According to the observations of Professor Tiedemann, the full size is acquired about the seventh or eighth year ; from this period there is little or no alteration, until a very advanced age, when in many cases a real diminution of bulk does actually appear to take place, accompanied by a corresponding contraction of the cavity of the cranium.

That the computations of anatomists concerning the proportion which the brain bears to the whole body should vary even more than their estimates of its absolute weight will not excite any wonder, if we reflect upon the very great differences which exist in the magnitude of the bodies of different individuals, altogether independent of any causes which could be supposed to exert an appreciable influence over the brain. We are, therefore, inclined to attribute but little importance to this branch of the enquiry ; nor do we think it necessary to enter upon any consideration of the facts adduced by Professor Tiedemann, in refutation of the old opinion, first promulgated by Aristotle, that the human brain is both absolutely and relatively larger than that of any other animal, because we believe that the fallacy of this assertion is universally acknowledged by anatomists, and because it is of little importance in determining the point in question. It may be well, however, to allude to the fact, which was first ascertained by Sœmmerring, and has been since confirmed by the researches of Blumenbach, Ebel, Cuvier,

Treviranus, Tiedemann, &c., viz. that the proportion which the brain bears to the cranial nerves, is greater in man than in any other animal, the orang not excepted; and also that the human cerebrum is the largest, when compared with the cerebellum, medulla oblongata, and medulla spinalis. This circumstance, when taken in connexion with the great superiority of man in regard to his mental capacity, the seat of which is undoubtedly in the brain, and his inferiority in many other functions of the nervous system, appears to us of the highest importance, and far more valuable in the present enquiry than any which has been hitherto mentioned.

Professor Tiedemann endeavours to show that the sensibility of different individuals, and of the same at different periods of life, is proportioned to the relation which the brain bears to the whole body. This may possibly be the case, though we cannot as yet believe it to be absolutely determined; but, even if true, it throws no light upon the question before us, for the very obvious reason that augmented nervous sensibility is by no means necessarily, nor indeed commonly, connected with increased mental capacity. Infants are possessed of a nervous system whose susceptibility is of the very highest degree, yet no one would contend that they exhibit any proofs of superior intelligence. The same is true of hysterical patients, who often, so far from being remarkable for more than ordinary mental powers, are even lamentably below the usual standard. "Emaciated persons," says our author, "are more sensitive than the corpulent;" but are they also more intellectual? It is needless to pursue the enquiry further.

II. *Weight of the Negro Brain.* With regard to the weight of the Negro brain, the observations of Tiedemann are much less satisfactory, on account of their very limited number. He commences with the assertion that the calculations of the weight of Negro brains by no means bear out the declaration of Camper, that the Negro, besides his acute facial angle, has also a smaller brain than the European; and the following are the grounds upon which he bases this opinion: Sœmmerring weighed the brain of a Negro boy, aged 14, and found it 3lb. 6oz. 3dr. apoth. In another, aged 20, but not full grown, it weighed 3lb. 9oz. 4dr., a greater weight, he remarks, than in many Europeans, better grown, and larger men. Mascagni found the brain of a Negress of twenty years to weigh 3½lb., which is more than he had ever met with among whites; but in a Negro the same observer found the brain only 1lb. 10oz. Sir A. Cooper gives the weight of the brain of a very fine Negro at 49oz. or 4lb. 1oz. A Negro examined by Tiedemann was twenty-five years of age, of short stature, and slender make. His brain, separated from the spinal cord at the medulla oblongata, weighed 2lb. 3oz. 2dr. He also measured the brains of two Negroes and a Bruges woman, in the Museum of Comparative Anatomy in the Royal Gardens at Paris, and declares them to be no smaller than the European, but he does not give the measurements.

Although from such limited data it is scarcely possible to draw anything like a fair conclusion, yet, as far as they go, they appear to us certainly not to support or confirm the inferences drawn from them by our author. The average weight of European brains, as stated by himself, varies from 3lb. 10oz. to 3lb. 11oz. in adult males, and from

3lb. 5oz. to 3lb. 8oz. in adult females, (pp. 17, 18); while of the four Negroes, whose weight can be readily compared with the above, because the scale of measurement is known, the average amounted to only 3lb. 5oz. 15gr., thus scarcely exceeding the lowest average for females. The *highest* weight of the European brains which he examined was 4lb. 11oz. 4dr.; the *highest* of the Negro 4lb. 1oz. The *lowest* male European was 3lb. 2oz. 20gr. apoth.; the *lowest* male Negro 1lb. 10oz. (Mascagni, scale unmentioned). It may be well to remark here that Professor Tiedemann's averages for European brains in general are considerably above those of most other observers: thus, for males, he exceeds Sir W. Hamilton by 5oz., Dr. Sims by 7oz., and Dr. Clendinning, by 7¾oz.; for females, also, there is an analogous difference. (Vide *Lond. Med. Gazette*, June 23, 1838.) But even this will not alter the state of the case, for the average of the above-mentioned Negroes falls much below even the smallest of these.

III. In the absence of sufficient opportunities for examining Negro brains, Professor Tiedemann next directs his attention to measuring the *cavum cranii* in the different races of mankind, and thus computing the size of their contents. For this purpose the following means were employed. The dry skulls were first weighed; they were then filled with millet-seeds, introduced through the foramen magnum, the escape of these by the other foramina and sutures being carefully guarded against. During the process frequent agitation was employed, in order that the seeds might lie closely packed. The full skull was then weighed, and its previously-ascertained weight deducted; the remainder gave, of course, the measurement of the cavity. In this manner several hundred skulls, from all nations of the earth were measured, either by himself or under his immediate inspection, few being submitted to this examination whose authenticity was in any degree doubtful. The results for each great division of mankind are given in separate tables; but these are far too long for insertion, and we shall therefore content ourselves with a simple analysis of them.

*Æthiopian race.* In 53 true Negroes the cavum cranii contained by weight from 54oz. 2dr. 33gr. to 31oz. 5dr. 16gr. In 4 Caffres it was between 43 and 37oz. In 7 Hottentots and Bosjemans, between 42 and 32oz. In 5 Mulattoes, between 48 and 34oz. In 12 Negresses it was between 38oz. 6dr. 30gr. and 24oz. 7dr. 39gr. In a female Caffre, 39oz. 1dr. In four Hottentot women, between 35 and 31oz. In a female Mulatto, 34oz. 6dr. 16gr.

*Caucasian race.* Of 186 males the cavum cranii contained from 57oz. 3dr. 56gr. (a Cossack from the Don,) to 27oz. 6dr. 30gr. (a Hindoo.) In 22 females it varied from 40oz. 6dr. 20gr. (Dutch,) to 28oz. 4dr. 24gr. (Hindoo.)

*Mongolian race.* In 46 males the cavum cranii varied from 49oz. 1dr. 22gr. (Esquimaux) to 13oz. 5dr. 24gr. (Baschkir.) It should be remarked, however, that in two other Baschkirs it was 44 and 40oz., so that this very small capacity cannot be looked upon as characteristic of that tribe. In 3 females it was between 36 and 31oz.

*American race.* In 31 males the cavum cranii weighed from 59oz. to 26oz. 1dr. 44gr. In 4 females, from 40oz. 5dr. 22gr. to 31oz. 43gr. The same tables also prove that the artificial flattening of the skull, which is so commonly practised among certain tribes of this race, has no effect in diminishing its capacity.

*Malay race.* In 98 males the cavum cranii varied from 49 oz. 1 dr. 45 gr. (inhabitant of the island Huaheine,) to 22 oz. 2 dr. (inhabitant of Amboyna.) In 10 females, it was from 41 oz. (Javanese,) to 19 oz. 2 dr. 29 gr. (Lascar.)

The whole of the facts ascertained on this point by M. Tiedemann are exhibited in a very clear manner in Tables, which are, however, too voluminous to extract. The following are the more important results deducible from them:

*Males.* The cavum cranii in the different races of mankind exhibits every variety, from 59 to 13 oz.; but by far the greater number are found between 42 and 32 oz. Thus,

| | | | |
|---|---|---|---|
| Of 69 | males of the Æthiopian race | 64 | were so circumstanced. |
| 186 | Caucasian | 143 | |
| 46 | Mongolian | 29 | |
| 31 | American | 21 | |
| 98 | Malay | 63 | |

A capacity of *more than* 42 oz. occurred in 5 Æthiopians, 42 Caucasians, 10 Mongolians, 7 Americans, and 21 Malays; and a capacity *under* 32 oz. occurred in only 1 Negro and 1 Caucasian, but in 3 Americans, 7 Mongols, and 14 Malays.

*Females.* The cavum cranii varies among females from 41 oz. to 19 oz., and is upon the whole smaller than in males. In the majority it was between 38 and 30 oz. viz.

| | | | |
|---|---|---|---|
| Of 18 | Æthiopian women | 16 | were so circumstanced. |
| 22 | Caucasian | 19 | |
| 3 | Mongolian | 3 | |
| 4 | American | 3 | |
| 11 | Malay | 7 | |

*More than* 38 *oz.* were only found in 1 Negress, 2 Caucasians 1 American, and 1 Malay; *less than* 30 *oz.* only in 1 Negress, 1 Cauca sian, and 3 Malays.

"The results of our enquiries," says Professor Tiedemann, in commenting upon these tables, " undeniably show that those anatomists and naturalists have fallen into error who have described the cranium of the Negro of a less capacity, and his brain smaller than that of Europeans and the other races." To a certain extent this inference is correct, the tables evidently showing that there is no such great inferiority in the Negro, as has been frequently asserted. At the same time, it is impossible for an unbiassed observer to fail being convinced of the fact, that the average capacity of the Æthiopian skull is somewhat lower than that of the European, and that a large size is considerably less common among them than among *any other races of mankind.* We forbear, for the present, entering more fully upon this subject, reserving our remarks until we have occasion to notice more particularly the state of intellectual development of this people.

IV. *Comparative measurements of the Brain and Spinal Cord of Negroes and Europeans.* There was no apparent difference in the external aspect, nor in the internal structure of the medulla spinalis of a Negro (Honoré), a man of small stature, whose body Tiedemann had an opportunity of examining. The following table exhibits a comparative view of its dimensions and those of the same parts in a male European of 5 feet 8 in. and in a woman of 5 feet.

| | Negro. in. | Negro. lines. | Europ. Man. in. | Europ. Man. lines. | Eur. woman. in. | Eur. woman. lines. |
|---|---|---|---|---|---|---|
| Length of whole cord from the pons variolii... | 14 | 11 | 17 | 3 | 14 | 10 |
| Breadth of medulla oblongata below the pons | | 10 | | 11 | | $10\frac{1}{3}$ |
| at decuss. of pyramids ................ | | $5\frac{2}{3}$ | | $6\frac{1}{4}$ | | $5\frac{1}{6}$ |
| of med. spin. in upper part of neck | | $5\frac{1}{3}$ | | $5\frac{1}{2}$ | | $5\frac{1}{2}$ |
| in lower part of neck .................. | | $6\frac{2}{3}$ | | $6\frac{3}{4}$ | | $6\frac{1}{3}$ |
| in middle dorsal region ............... | | $4\frac{1}{2}$ | | 5 | | $4\frac{1}{3}$ |
| in lower dorsal region ................ | | $5\frac{1}{3}$ | | $5\frac{2}{3}$ | | $5\frac{1}{4}$ |

The slight differences observed here may be evidently traced to variations in the whole size of the body; and it is of importance to notice that the Negro differs from the others on the side of *deficiency*, not of *excess*, because, as we shall see afterwards, in the orang, to which he has been so often compared, the case is precisely reversed.

The two next tables in the text exhibit the measurements of the cerebellum and nodus encephali (pons variolii) in 4 Negroes and 9 Europeans; we have reduced them into one, giving merely the averages, which are quite sufficient for our purpose.

| | Negro. in. lines. | European. in. lines. |
|---|---|---|
| Greatest breadth of the cerebellum........................ | 3 $5\frac{3}{4}$ average of 4 | 3 $7\frac{8}{9}$ average of 9 |
| Long diameter of cerebellum in the middle............... | 2 $4\frac{7}{8}$ average of 4 | 2 $5\frac{1}{12}$ average of 6 |
| Breadth of the nodus encephali between the fifth pair of nerves........................................ | 1 $1\frac{1}{2}$ in 1 case | 1 $1\frac{83}{96}$ average of 8 |
| Long diameter of the same ............................ | 0 $10\frac{1}{3}$ in 1 case | 0 $14\frac{1}{18}$ average of 6 |

Here, again, it should be particularly remarked, that in the Negro these parts are rather of an *inferior* than *superior* magnitude.

The following table represents the comparative measurements of the cerebrum in 4 Negroes, 7 European males, and 6 European females; it is reduced from three tables, which are given in the original, by taking the averages as before.

| | | in. | lines. |
|---|---|---|---|
| Average length of brain in | 4 Negroes.................. | 5 | 11 |
| | 7 European males....... | 6 | $2\frac{1}{7}$ |
| | 6 European females ..... | 5 | $10\frac{1}{2}$ |
| Average greatest breadth in | 4 Negroes ............... | 4 | $8\frac{1}{6}$ |
| | 7 European males ..... | 5 | $1\frac{1}{7}$ |
| | 3 European females ... | 5 | $4\frac{1}{3}$ |
| Average height of brain in | 3 Negroes.................. | 2 | $11\frac{1}{3}$ |
| | 7 European males ...... | 3 | 4 |
| | 4 European females .... | 2 | $9\frac{1}{2}$ |

There is clearly, then, notwithstanding the assertions of Tiedemann, a very considerable inferiority of size in the cerebrum of the Negro; and this perfectly coincides with the inferences we have already drawn from the measurements of the cavum cranii. In the arrangement of its parts and in their more intimate structure, the Negro brain is precisely similar to that of the European: the only perceptible difference being that the convolutions were somewhat broader in the anterior lobes, and also more symmetrically arranged in the two hemispheres, than is commonly observed. The cerebellum was entirely concealed, and partially overlapped posteriorly by the cerebrum. In apes the opposite obtains.

V. *Has the Negro thicker or larger Nerves than the European?*—Sœmmerring, who, of all anatomists, has paid most attention to this subject, has laid it down as a rule that in the Negro the cranial nerves are somewhat larger than in the European, and that this difference is most remarkable in the olfactory, the optic, and the fifth pair. But Professor Tiedemann not having observed this to be the case in the four brains which he examined, considers himself entitled to deny the existence of any such difference whatever. We must, however, confess that there appear to us very insufficient grounds for such a conclusion; and in the absence of more complete and satisfactory evidence, we feel much inclined to adhere to the old opinion, which is, moreover, supported by the larger size of the foramina in the base of the skull, through which these nerves pass, and the much greater development of the organs of sense in the Negro. "Nature," says Dr. Prichard, "seems to have made, if we may use the expression, a more careful provision for the perfect and full development of the sensitive power. The orbits are large and capacious; the cavity of the nose has a remarkable amplitude in the Negro, and all the parts which are subservient to the sense of smelling have a very perfect conformation. The upper turbinated bones are large and finely convoluted, presenting an extensive surface for the expansion of the nasal membranes. The passages of the posterior nostrils are wider in the Negro than in the white man. The African has accordingly, as it has been often remarked, a very acute perception of odours. It has been asserted that the Negroes of the Antilles can distinguish in pursuit the track of white and black people by the sense of smell."

VI. *Does the Brain of the Negro bear a greater resemblance to that of the Orang-outang than the European?* The result of M. Tiedemann's dissections of two individuals of this species, one from Java, the other from Africa, and preserved in the Hunterian Collection, Royal College of Surgeons, London, have led him to the conclusion that the brain of apes and orangs differs from the human in the following respects: 1. The cerebrum is absolutely and relatively to the mass of the body smaller, shorter, narrower, and more flattened. 2. It is smaller in relation to the nerves. 3. The cerebral hemispheres bear a smaller proportion to the spinal cord, medulla oblongata, cerebellum, and corpora quadrigemina. 4. There are fewer convolutions and shallower sulci. In all these it differs from the Negro as well as from the European, the only point of greater resemblance between them being the more symmetrical arrangement of thc convolutions. How much less the relative size of the brain to the body is in orangs than in man is well illustrated by the fact that in a large pongo from Borneo, the cavum cranii measured only 11 oz. 7 dr., which is even less than in congenital idiots.

VII. As a final result of these investigations, Professor Tiedemann believes himself entitled to draw the following conclusions: 1. That the brain of the Negro is, in general, or on an average, as large as that of the European and other races of mankind. (In Europeans and Malays a brain *below* the average size is more common than in Negroes.) 2. That the cranial nerves of the Negro are not, as Sœmmerring imagined, larger than those of the European. 3. That the medulla spinalis, the medulla oblongata, the cerebellum, and cerebrum of the Negro exhibit no difference from the European, either in their external appearance or internal structure, excepting that the hemispheres of the cerebrum are somewhat smaller. 4. That the brain of the Negro bears no more resemblance to that of the orang than the European does, excepting the more symmetrical arrangement of the convolutions in the two hemispheres. But this cannot be considered as established.

In reflecting upon these inferences and the data upon which they are founded, we have been unavoidably led to the opinion that the learned author of the paper before us has allowed his philanthropic feelings to betray him into the very common but most dangerous error of hasty generalization. We have seen that the few Negro brains submitted to examination exhibited an average size considerably inferior to that of Europeans, and that the measurements of the cavum cranii bore witness to the same fact; the conclusion from which is inevitable, and directly opposed to the inference drawn by our author. And it should be particularly observed here that the *average* size is all with which we are concerned, not the fact that in a few instances the brain reaches a more than ordinary magnitude; because it is evident that if the question is to be determined at all in this way, it must be from the *general rule*, not from the *exceptions*. But we may very properly enquire in this place whether the method adopted in this investigation is calculated to lead to the results expected, whether the magnitude of the whole encephalon is a measure of the intellectual capacity of the possessor? On this point we would again refer our readers to the very valuable observations of Dr. Combe, inserted in our Fifth Vol., p. 385, believing that they exhibit in a most satisfactory manner the errors into which Professor Tiedemann has fallen. For whether we adopt wholly or only partially the views of the phrenologists regarding the subdivision of the brain into organs, we must acknowledge that it seems to us sufficiently established that the higher powers of the intellect are more decidedly proportioned to the magnitude of the anterior lobes of the cerebral hemispheres than to that of any other portion of the cranial contents; and in this respect, by Tiedemann's own confession, the Negro is very deficient. At the same time, it is but justice to observe that the same measurements which have led to this conclusion also prove that the organs which, if phrenologists be correct, are chiefly connected with mere animal propensities, do not exhibit any excess of development, and that if the Negro brain is, in some respects, *inferior* to that of the European, it is in the same particulars much more decidedly *superior* to that of apes.

On the whole, then, we feel considerable doubts as to the possibility of determining the point in question by any such researches as those which we have been engaged in examining, partly on account of the difficulty of obtaining sufficient data for the purpose, and partly because the evidence thus procured is of a very uncertain character. The same objections, however, do not by any means apply to the other statements advanced by our author in favour of the Negro's capability for moral and intellectual improvement. To these we are inclined to attribute far more weight, because they are based upon the results of actual experience, and are unconnected with any theory or hypothesis whatever.

That even the external characteristics of the Æthiopian variety of mankind, as laid down by many writers, are mere caricatures, drawn from partial observation of exaggerated peculiarities, is now sufficiently established; and precisely the same may be said with regard to their intellectual and moral capabilities. The picture drawn by those who have only been acquainted with this unfortunate people, in the degraded position of slaves, and which portrays them as utterly vicious, malignant, perverse and faithless, irretrievably subdued by indolence, incapable of improvement, and scarcely even deserving a place among rational creatures, is so palpably overcoloured, so evidently the result of prejudice and imperfect observation, as to deceive none who will examine the subject for themselves. The accounts of those enterprising travellers who have penetrated into the interior of Africa are well known to be altogether different. These represent the African to possess, in full vigour, all the intellectual and moral qualities of his white brethren. It seems, however, to have been too generally overlooked by all parties, that there are distinct tribes among Negroes as among Europeans; and that, while some (those formerly most known) are very inferior, others, more recently discovered, especially in the interior of Africa, are proportionably superior. The former clans have low heads and smaller brains, while the latter approach, both in brain and feature, the European standard.

Another point of great importance, as influencing mental manifestations, has been entirely overlooked by Tiedemann and most others, viz. the *quality* of constitution or temperament. Generally speaking, the European is finer in fibre than the Negro, and hence, with otherwise equal powers, the former would be more susceptible of polish and refinement. Why should all Negroes be alike, any more than Europeans? The French brain is smaller than the English, as the French hatters experienced when they had to make blocks expressly for the English soldiers. This remark applies to Dr. Sims, and nearly all others who have weighed brains. How could the weight be uniform, when it is palpable to the eye that, taken generally, both Irish and French brains are smaller than English and Scotch; and, in some districts, all these are inferior to German and Swiss? But then the Swiss, with their large heads, have slow, heavy temperaments, and are, consequently, frequently inferior in actual performance to those with feebler but livelier powers. In this, as in every other enquiry, we must look about us, on all sides, if we would find truth; we must not follow any one path only, merely because we have chanced to hit upon it. In investigating the physical and intellectual characters of races as of individuals, we must take Nature as she is; and, with the more philosophic phrenologist, be careful not to mix the front and back, the top and base of the head, and one temperament with another, into one mass, and call it "fact."

In bringing these remarks to a conclusion, we feel it necessary to make a few observations in regard to the manner in which Professor Tiedemann has performed his self-imposed task. In perusing the work before us, we could not avoid being forcibly struck with and much grieved by the evident spirit of partisanship in which the whole enquiry has been conducted. Every circumstance, however trivial, which in any degree favoured his manifestly preconceived views, is largely dilated upon, and most fully credited; while facts of an opposite tendency are carelessly slurred over, or declared to be fallacious. To give an example: In comparing the brains of the Negro and the Orang, he remarks, that there appears to be one point of resemblance between them, which does not exist in the European brain, viz. the more symmetrical arrangement of the convolutions in the two cerebral hemispheres; but he immediately adds, "whether this mark is constant I cannot determine, because I have only examined four Negro brains." Yet, though the number of observations are precisely the same, he finds no difficulty whatever in founding upon them the general rule, that the cranial nerves of the Negro are neither thicker nor larger than those of the European. This is not the spirit in which a philosophical enquiry should be conducted; and it is truly melancholy to behold so old and experienced an observer falling into an error which should at least be confined to novices alone. On the whole, we do not think that this publication is by any means calculated to extend the learned author's reputation. It can boast of little originality, excepting in the method applied to elucidate the proposed questions; and this, for reasons already stated, we do not look upon as satisfactory. And although few could read his memoir without feelings of admiration and pleasure, at the heartfelt warmth and enthusiastic zeal with which he advocates the cause of an oppressed people, yet we believe that the majority would rise from the perusal with the impression that the facts and arguments brought forward, while they certainly exhibit the measureless superiority of the Negro over the ape, yet in a great measure, if not entirely, fail to establish the so eagerly contested point of his *perfect equality* with his more fortunate European brethren.

---

